# Effects of an extra Trp113Tyr substitution on yeast D-amino acid oxidase variant

**DOI:** 10.1186/2193-1801-4-S2-P6

**Published:** 2015-11-27

**Authors:** Kin-sing Wong, Wing-ping Fong, Paul Wai-kei Tsang

**Affiliations:** Racing Laboratory, The Hong Kong Jockey Club, Hong Kong; School of Life Sciences, The Chinese University of Hong Kong, Hong Kong; School of General Education and Languages, Technological and Higher Education Institute of Hong Kong, Hong Kong

## Background

β-Lactam antibiotics (cephalosporins and penicillins) are used in clinical practice to combat microbial infections. Commercial cephalosporins are all semi-synthetic and chemically derived from 7-aminocephalosporanic acid (7-ACA), a cephem nucleus that is traditionally produced by multi-step chemical reactions from cephalosporin C (CPC). However, the procedures are environmentally damaging, costly, and low yield [1]. The increasing annual demand for semi-synthetic cephalosporins and the concept of environmental sustainability have created a dire need to develop a 'greener' production of 7-ACA. 7-ACA can be enzymatically produced from CPC by a two-step process that involves D-amino acid oxidase (DAAO) and glutaryl-7-aminocephalosporanic acid (GL-7-ACA) acylase. Recently, we characterised the highly active and thermostable *Trigonopsisvariabilis* DAAO (TvDAAO) variant F54Y for oxidative deamination of CPC. A single amino acid substitution at the 54th position of TvDAAO from phenylalanine (F) to tyrosine (Y) resulted in a 6-fold improvement in catalytic activity towards CPC and in thermostability after heat treatment at 55°C [2]. The potential application of the TvDAAO F54Y variant in the antibiotics industry as a simple and efficient biocatalyst was evaluated [3]. To further elucidate the structural basis of the catalytic activity and thermostability of TvDAAO F54Y, we investigated the role of the 113th amino acid residue (tryptophan, W113) that locates near the substrate binding cleft.

## Methods

The W113 (tryptophan) residue of the TvDAAO F54Y variant was converted to tyrosine by the overlapping polymerase chain reaction using mutagenic primers. The mutations in the resultant TvDAAO variant, TvDAAO F54Y/W113Y, were confirmed by automated DNA sequencing. The variant was expressed in *Escherichiacoli* BL21(DE3)pLysS. *E. coli* cells were harvested by centrifugation (2,400 g, 15 min) at 4°C and lysed in ice-cold 20 mM sodium phosphate (pH 7.0) supplemented with 1 mM phenylmethylsulphonyl fluoride by sonication (ten cycles, 15 sec each, at 15 sec intervals). The cell-free extract was clarified by centrifugation (13,000 g, 30 min) at 4°C, filtered through a 0.20 μm nylon membrane, and purified by a diethylaminoethyl-cellulose column and a HiLoad 16/60 Superdex 200 prep grade gel filtration column. Purity of the enzyme was analysed by sodium dodecyl sulphate-polyacrylamide gel electrophoresis, and its concentration was determined by Bradford assay. The catalytic activity of the purified enzyme was determined by measuring the formation of GL-7-ACA at 22°C with agitation [3]. One mL of the reaction mixture contained 20 μg TvDAAO F54Y/W113Y variant, 5 mM cephalosporin C, and 50 mM sodium phosphate (pH 7.5). The amount of GL-7-ACA liberated was quantified by high performance liquid chromatography equipped with a C-18 reverse phase column, using 6% acetonitrile in 50 mM potassium phosphate (pH 7.5) as the mobile phase. Thermostability of the purified enzyme was evaluated by measuring the residual catalytic activity after heat treatment at 55°C for 30 min.

## Results

The TvDAAO F54Y/W113Y variant was prepared and purified to homogeneity. Catalytic activity was increased by ~10% with respect to the original TvDAAO F54Y variant, but was deactivated by heat treatment.

## Conclusions

The W113 of TvDAAO plays a pivotal role in thermal deactivation. W113Y substitution may alter the local spatial arrangement of the substrate binding cleft, and hence increase the vulnerability of the nearby oxidative-sensitive C108 [4], promoting deactivation of the enzyme at elevated temperatures.
